# Tracking Mycoviruses in Public RNAseq Datasets of *Malassezia*: Three Original Totiviruses Revealed

**DOI:** 10.3390/v15061368

**Published:** 2023-06-13

**Authors:** Fatima Boulanouar, Stéphane Ranque, Anthony Levasseur

**Affiliations:** 1 Aix-Marseille Université (AMU), UMR VITROME, IRD, APHM, Faculté de Médecine, 13005 Marseille, Francestephane.ranque@ap-hm.fr (S.R.); 2 Aix-Marseille Université (AMU), UMR MEPHI, IRD, APHM, Faculté de Médecine, 13005 Marseille, France; 3 IHU Méditerranée Infection, 13005 Marseille, France; 4 Institut Universitaire de France, 75005 Paris, France

**Keywords:** *Malassezia*, mycovirus, *Totivirus*, transcriptomes, dsRNA, fungal evolution, fungal host, skin disorders

## Abstract

Mycoviruses are viruses that selectively infect and multiply in fungal cells. *Malassezia* is the most abundant fungus on human skin and is associated with a variety of conditions, including atopic eczema, atopic dermatitis, dandruff, folliculitis, pityriasis versicolor, and seborrheic dermatitis. Here, we conducted mycovirome studies on 194 public transcriptomes of *Malassezia* (2,568,212,042 paired-end reads) screened against all available viral proteins. Transcriptomic data were assembled *de novo* resulting in 1,170,715 contigs and 2,995,306 open reading frames (ORFs) that were subsequently tracked for potential viral sequences. Eighty-eight virus-associated ORFs were detected in 68 contigs from 28 Sequence Read Archive (SRA) samples. Seventy-five and thirteen ORFs were retrieved from transcriptomes of *Malassezia globosa* and *Malassezia restricta*, respectively. Phylogenetic reconstructions revealed three new mycoviruses belonging to the *Totivirus* genus and named Malassezia globosa-associated-totivirus 1 (MgaTV1); Malassezia restricta-associated-totivirus 1 (MraTV1) and Malassezia restricta-associated-totivirus 2 (MraTV2). These viral candidates extend our understanding of the diversity and taxonomy of mycoviruses as well as their co-evolution with their fungal hosts. These results reflected the unexpected diversity of mycoviruses hidden in public databases. In conclusion, this study sheds light on the discovery of novel mycoviruses and opens the door to study their impact on disease caused by the host fungus *Malassezia* and globally, their implication in clinical skin disorders.

## 1. Introduction

High-throughput sequencing technologies have revolutionised our ability to unravel the molecular world. Transcriptome projects result in vast amounts of RNA sequences, which are particularly useful for delivering targeted information about gene expression and improving the annotation of genome projects. Nevertheless, the RNA content of a cell also comprises parasitic molecules, such as the genomes of RNA viruses and their replication intermediates. Although the first report on mycoviruses was published over 60 years ago by Hollings, they have continued to be isolated from all major taxonomic groups of fungi [1]. Typically, mycoviruses are either encoded by positive or negative sense double-stranded (ds) or single-stranded (ss) RNA. To date, only one single-stranded DNA (ssDNA) mycovirus with a circular genome has been described from the plant pathogenic fungus *Sclerotinia sclerotiorum* [2]. Double-stranded DNA (dsDNA) mycoviruses have not yet been discovered. According to the International Virus Taxonomy Committee (ICTV), fungal viruses have been taxonomically classified into 27 formal families. Most mycoviruses have dsRNA genomes and are grouped into ten families: *Amalgaviridae*, *Chrysoviridae*, *Endornaviridae*, *Fusagraviridae*, *Megabirnaviridae*, *Partitiviridae*, *Polymycoviridae*, *Quadriviridae*, *Spinareoviridae*, and *Totiviridae*. Linear, positive-sense ssRNA mycoviruses are classified into eleven families: *Alphaflexiviridae*, *Barnaviridae*, *Botourmiaviridae*, *Deltaflexiviridae*, *Endornaviridae*, *Gammaflexiviridae*, *Hypoviridae*, *Metaviridae*, *Mitoviridae*, *Narnaviridae* and *Tymoviridae.* The families *Mymonaviridae*, *Phenuiviridae* and *Rhabdoviridae* have (-)ssRNA. The *Metaviridae* and *Pseudoviridae *families have (+) ssRNA-RT genomes [3,4]. The family *Genomoviridae* have ssDNA genomes. Mycoviruses have no known extracellular mode of transmission but are efficiently transmitted to other fungal cells through two major intracellular transmission pathways: vertical transmission from fungal mycelium to spores through the cytoplasm during spore formation, and horizontal transmission between different fungal individuals which are vegetatively compatible via hyphal anastomosis [5]. However, the transmission of hypoviruses between two vegetatively incompatible strains of *S. sclerotiorum* has been confirmed by Xiao et al. [2]. Mycoviruses can reduce the ability of fungi to cause diseases by altering the host phenotype, sporulation efficiency and growth rate, and act as a hypovirulence factor [6]. They can be used as biological control agents, as demonstrated by the successful use of Cryphonectria hypovirus 1 (CHV1) for the control of chestnut blight disease in Europe [7,8,9]. Conversely, mycoviruses can also confer hypervirulence by promoting sporulation, aggressiveness and growth [10], or may permit the production of extracellular antifungal toxins [11]. These mycoviruses could confer fitness advantage by toxin-producing fungal strains as reported for *Saccharomyces cerevisiae* [12] and *Ustilago maydis* [13]. Furthermore, Thapa et al. reported the case of mycoviruses infecting the filamentous fungus *Pseudogymnoascus destructans* [14]. *P. destructans* provokes white-nose syndrome in bats causing 5 million deaths in North America. Interestingly, all *P. destructans* isolates from North American bats harbour a mycovirus contrary to the European and Asian isolates known to be resistant to the endemic fungus. Generally, mycoviruses are detected by sequencing cDNA generated from dsRNA fragments isolated from fungi [9]. Rapid advances in and application of NGS techniques have increased the volume of sequencing data available in public databases, which are freely available, such as the National Center for Biotechnology Information (NCBI) Short Read Archive (SRA) [15]. Transcriptomic data offer the possibility of searching for viruses in various fungal samples without *a priori* knowledge of the viral infection [16]. *Malassezia* represents the most dominant fungal genus on the surface of the human skin, and species in this group are responsible for various skin diseases including atopic dermatitis, dandruff, and seborrheic dermatitis, as well as systemic infections. To date, eighteen species of *Malassezia* have been identified [4] and the sequence for seventeen of these genomes is publicly available. Recently, a few mycoviruses belonging to the *Totiviridae* were isolated and identified in some *Malassezia* species. These include two distinct viruses: MrV40, which affects fungal host cell physiology and induces a TLR3-mediated inflammatory immune response in bone marrow-derived dendritic cells, indicating that a viral component is likely to contribute to *Malassezia* pathogenicity [4], and MsMV1, which also induces an immunological response. MsMV1 infection causes an upregulation of a large number of ribosomal genes and the production of interferon-β in cultured macrophages [17]. In the literature, the presence of mycoviruses in some *Malassezia* species remains scarce. In the present study, our objective was to explore transcriptome sequencing data to unravel and to track mycoviruses in public databases. Thus, we carried out an in silico mycovirome study to identify mycoviruses infecting fungi of the *Malassezia* genus, using the publicly available SRA datasets in NCBI. A bioinformatic pipeline was designed for the identification of viral-like sequences. Evolutionary studies were carried out for all candidate ORFs of mycoviruses in order to disclose their taxonomic assignment.

## 2. Materials and Methods

### 2.1. Datasets

One hundred and eighty-four publicly available RNA-Seq libraries, including four *Malassezia* species: *M. globosa*, *M. pachydermatis*, *M. sympodialis*, and *M. restricta* (see Supplementary Table S1), were analysed for the presence of RNA viruses.

### 2.2. Pipeline for Identification of Viral-like Sequences

**Figure 1:**
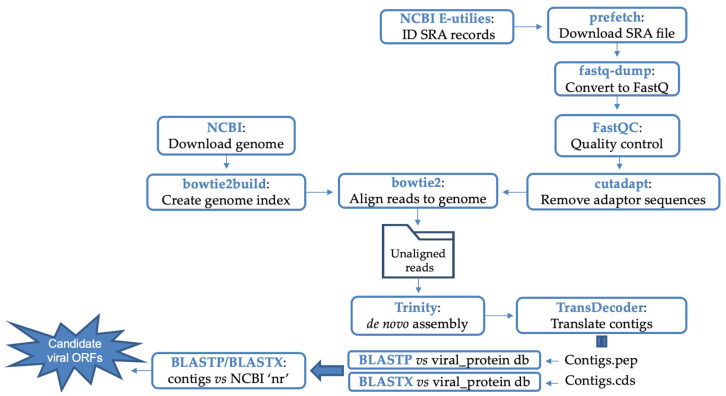
Flowchart of the main steps and bioinformatics tools used in this study.

The computational steps for identifying viral sequences began with downloading all available *Malassezia* raw reads files from the SRA database accessed on 11 february 2021 (https://www.ncbi.nlm.nih.gov/sra). The SRA Toolkit programme, fastq-dump (v2.9.1), was used to download and convert the SRA file to FastQ format. Quality control and cleaning of the reads was performed with FastQC [18] and Cutadapt [19], respectively. Raw reads were mapped to a genome index created from four reference genomes of *Malassezia* using Bowtie2 (v2.2.5) [20]. Unmapped reads were collected and used for *de novo* assembly using Trinity (v2.4.0) [21] with default settings, and resultant contigs were analysed to predict open reading frames (ORFs) using TransDecoder (v5.5.0) [22]. A formatdb blast was used to generate a Blast database containing all known virus protein sequences extracted from GenBank. The predicted ORFs were queried against the viral proteome database by BLASTP (v2.8.1+). Significant hits against the virus database were then re-examined using BLASTP and BLASTX searches against non-redundant (‘nr’) database within NCBI, to remove sequences derived from hosts and other organisms such as eukaryotes and bacteria (Figure 1). The MEME suite (v5.5.0) was used to compare the conserved motifs between sequences [23]. All data were submitted at EMBL under the bioproject number PRJEB62036.

### 2.3. Phylogenetic Tree Construction

Phylogenetic analysis was carried out by selecting protein sequences with an e-values threshold equal to e-03 from the ‘nr’ database within NCBI. Multiple sequence alignment was produced using CLUSTALW programmes (BLOSUM matrix, 10 gap opening, 0.1 gap extension) [24]. Maximum likelihood-based phylogenetic analyses were performed using RAxML, with 1000 bootstrap replicates and the PROTCAT amino acid substitution model [25]. The final trees were visualised using iTOL (v5) [26].

## 3. Results

### 3.1. Mycoviruses in Malassezia spp. Transcriptomes

To identify mycoviruses in *Malassezia* spp., we analysed the publicly available RNA-Seq data sets of *M. globosa* under BioProject Accession number PRJNA658716, PRJNA286710, PRJNA18719; *M. sympodialis* under BioProject Accession number PRJEB13164, PRJNA593722, PRJNA626605; *M. restricta* under BioProject Accession number PRJNA513301, PRJNA577935, PRJNA593855; and *M. pachydermatis* under BioProject Accession number PRJNA485318. Initially, a total of 2,568,212,042 paired-end reads were mapped against the genomic DNA sequences of the four *Malassezia* reference genomes, *M. globosa* under RefSeq number GCF_000181695.1; *M. sympodialis* under RefSeq number GCF_000349305.1; *M. restricta* under RefSeq number GCF_003290485.1; *M. pachydermatis* under RefSeq number GCF_001278385.1. Unmapped reads were collected and used for *de novo* assembly and subsequent ORFs detection. The resulting ORFs were subjected to a BLASTP search against the viral proteome database, resulting in 2,995,306 ORFs with an e-value cut-off of 1e-10. After removing sequences derived from hosts and other organisms, 88 putative viral ORFs were identified, with lengths ranging from 120 to 767 aa, in a total of 68 contigs assembled from 28 of the 194 SRA samples analysed. Seventy-five of them were found in *M. globosa* and 13 in *M. restricta* transcriptomes. No viruses could be detected in the available *M. sympodialis* and *M. pachydermatis* transcriptomes. The putative viral ORFs were homologous to known viral proteins, including RNA-dependent RNA polymerase (RdRp) (28 ORFs), capsid protein (CP) (56 ORFs), and polyprotein (four ORFs) belonging to four different viruses. Contigs had both CP and RdRp proteins (22 contigs), CP and polyprotein (2 contigs), or a single CP (35 contigs), or a single RdRp (7 contigs), or a single polyprotein (2 contigs). The four viruses identified from the transcriptomes were derived from dsRNA genomes and included members of *Totiviridae* family. An in-depth analysis and characterisation of each of the 88 viral sequences is described in Supplementary Table S2. By aligning the different ORFs, at the nucleotide level, all the RdRp sequences identified in *M. globosa*, as well as the CP sequences in *M. globosa* and the polyprotein sequences in *M. restricta*, were identical. However, the alignment of CP sequences in *M. restricta* displayed three groups of sequences, which implies the identification of three distinct viral species associated with *Malassezia restricta*. Comparison of Chargaff’s ratio (GC%) between identified sequences and closely related species showed that sequences identical to the viral species Totiviridae sp., DTtV1 and MrV40L have similar GC% with their closely related species (Standard Deviation (SD) = 2.05). Meanwhile, widely different GC% were observed between sequences identical to the viral species EnaT7 and those closely related to it (SD = 7.30) (Supplementary Table S3). The functional annotation in these ORFs was also confirmed at the structural level using PHYRE2 (Supplementary Table S4).

### 3.2. Assignment of Identified Sequences to Their Taxonomic Group

In the family *Totiviridae*, species demarcation criteria are not absolute. However, less than 50% sequence identity at the protein level generally reflects a species difference as it was proposed by the ICTV within the *Totivirus* genus [27]. Based on these criteria, we proposed the two following names: (i) Malassezia globosa-associated-Totivirus 1 (MgaTV1), with Erysiphe necator-associated-Totivirus 7 (QJW70337.1) as the closest relative, RdRp aa identity [46.87–50.55%] and Totiviridae sp. the closest relative CP aa identity [39.43–42.58%]; and (ii) Malassezia restricta-associated-Totivirus 1 (MraTV1), which has [42.24–43.16%] CP aa identity and [49.64–60.81%] polyprotein aa identity to its closest relative Totiviridae sp. (QJT93774.1), and (WAK77294.1). The closest relative of MraTV2, the second virus identified in *M. restricta*, was Dali Totiv tick virus 1 (UYL95681.1) at [83.69–84.62%] CP aa identity finding in the tick metagenome. Despite the higher identity percentage, we suggest the genome be given the provisional name Malassezia restricta-associated-Totivirus 2 (MraTV2). We hypothesised that the occurrence of this virus in the tick metagenome could be associated with *Malassezia* colonization/infection of the ticks. Notably, this is the first identification of the viruses described above in *Malassezia*. The third virus found in *Malassezia restricta* belongs to a species previously described as MrV40L [4], sharing 100% RdRp aa identity with accession QJA42331.1 and 100% CP identity with accession QJA42330.1. This virus serves as an internal control for the virus discovery pipeline, as it was previously identified via a different approach from the same transcriptome SRA file. These congruent results support the validity of our pipeline (Figure 1).

### 3.3. Phylogenetic Analysis and Characterisation of Conserved RdRp and CP Regions of Totiviruses

**Figure 2:**
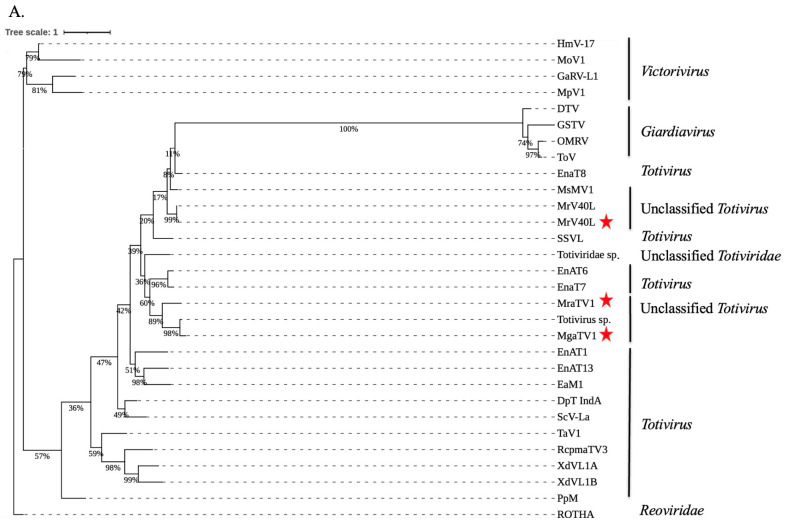

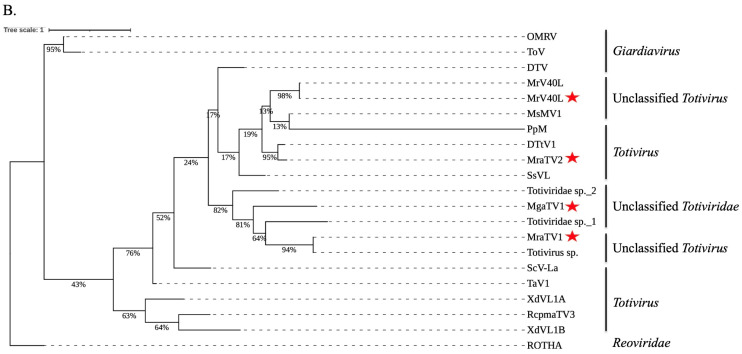
Phylogenetic reconstruction based on the sequences of RdRP (**A**) and CP (**B**). Mycoviruses from the genus *Totivirus, Giardiavirus, Victorivirus* and unclassified *Totivirus* families were represented. Human Rotavirus A (ROTHA) served as an outgroup. Candidate sequences were labelled with a red star. Virus names have been abbreviated; full names and accession numbers for protein sequences are in Supplementary Table S5. Phylogenetic trees were generated using Raxml under the model PROTCAT of amino acid substitution. Scale bar represents the number of substitutions per site. RDRP, RNA dependent RNA polymerase; CP, capsid protein.

To study the evolutionary relationships between candidate ORFs and mycoviruses from the *Totivirus*, *Giardiavirus*, *Victorivirus* and unclassified *Totivirus* genera, we used maximum-likelihood phylogenetic analyses of the RdRP and CP amino acid sequences (Supplementary Table S5). Based on genomic features, i.e., on the non-segmented organisation of their genome and the apparent structural similarity between the different ORFs and the proteins of the ScV-L-A species (the prototype virus of the genus *Totivirus)*, we assumed that these candidate viruses belong to the *Totiviridae* family. Phylogenetic analysis performed using RdRps sequences showed a clustering of the newly found MgaTV1 and MraTV1 with mycoviruses of unclassified *Totivirus* and *Totivirus* genus. The MrV40L identified in this study was clustered with the previously described MrV40L by Park et al. as expected (Figure 2A). Moreover, phylogenetic analysis of the CP sequences obtained from RNA-seq confirmed that the Mrv40L was also phylogenetically similar to the previously identified MrV40L [4]. However, the newly putative CP found MgaTV1, MraTV1, and MraTV2 cluster within members of unclassified *Totiviridae*, unclassified *Totivirus* and the genus *Totivirus,* respectively (Figure 2B).

**Figure 3:**
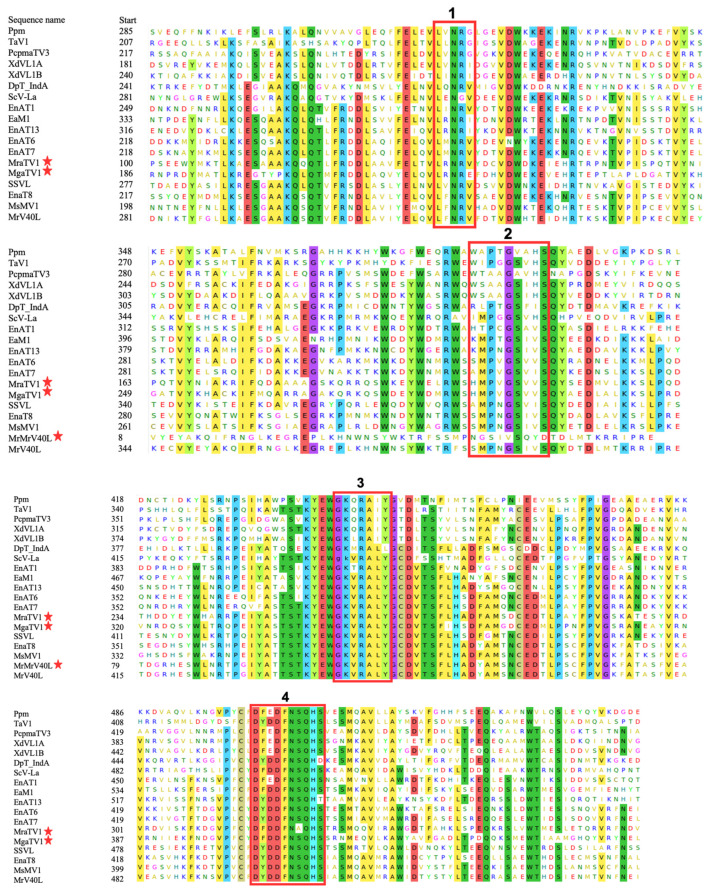

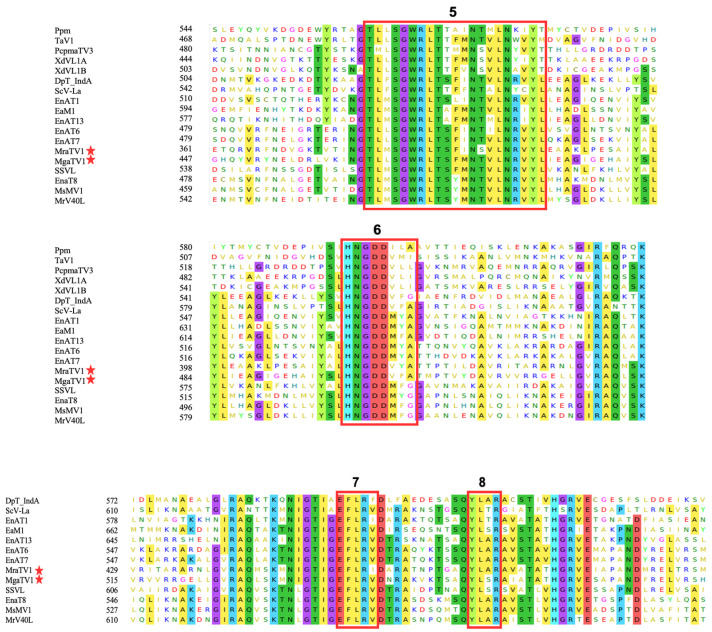
Conserved motifs identified in the RdRP domain of the genus Totivirus based on the multiple sequence alignment using MEME suite [23]. Candidate sequences were labelled with a red star. As with other Totiviruses, eight conserved motifs were retrieved. These conserved motifs were labeled **1**–**8** as RdRP associated motifs described above [28].

To confirm the presence of functionally conserved motifs of RdRp and CP in candidate viruses, we further analysed and compared eight RdRp motifs which are commonly found in totiviruses [28]. The eight conserved motifs were found within RdRP of the newly found MgaTV1 and MraTV1, supporting their classification as a *Totivirus*, whereas only two of these motifs were observed in MrV40L, owing to the reduced size of the candidate sequence (Figure 3). In addition, an alignment of the sequences of CP with their closest homologues using the MEME suite [23] revealed the existence of eight conserved motifs in most CP of the analysed species (Figure S1).

## 4. Discussion

Most mycovirus species have been discovered by isolating and sequencing dsRNA fragments from fungal extracts. However, the recoverability of dsRNA replication intermediates from ssRNA viruses does not seem to be as efficient as that of dsRNA genomic RNA. This could be the reason for the under-reporting of ssRNA mycoviruses in various studies. Transcriptome analyses use both single- and double-stranded RNA, and this may be a more sensitive approach to detecting and discovering the two RNA types, ssRNA and dsRNA. In the current study, we identified mycoviruses in silico within public *Malassezia* transcriptome datasets. Two recent in silico studies have been conducted on available fungal transcriptomes, but they differed in both methods and fungal species studied [16,29]. Our study focused on mycoviruses infecting the genus *Malassezia*. We analysed the 194 available *Malassezia* transcriptomes of four *Malassezia* species. Yeonhwa Jo et al. [29] have focused on 126 fungal transcriptomes from 11 fungal phyla, and Gilbert et al. [16] have studied Pezizomycotina.

Although the presence of dsRNA and virus-like particles has been reported in some *Malassezia* species [4,17], data on *Malassezia* mycoviruses remain scarce. Instead of only using the viral RdRp sequences, as previously [16], we conducted a BLASTP search on all available viral proteins to enhance our capacity to detect virus-associated contigs. We thus detected a wide range of mycoviruses encoding various viral proteins. After discarding contaminants sequences, we identified 86 novel genomic segments derived from dsRNA genomes putatively associated with three novel Totiviruses: MgaTV1, MraTV1, and MraTV2, which were identified in *M. globosa*, or *M. restricta.* All MgaTV1 sequences displayed less than 50% aa sequence identity to the closest known Totiviruses (Supplementary Table S1), thus meeting the criteria for a new virus species within the *Totivirus* genus, in keeping with the ICTV [30]. The CP sequences of MraTV1 showed less than 50% sequence identity with the closest known *Totivirus*, but two of the four polyproteins (RT-like superfamily) sequences exhibited more than 50% identity with the closest known *Totivirus*. Nonetheless, the criteria for defining a new virus species were met, because both the CP sequences and the other two polyproteins (RT-like superfamily) shared less than 50% identity. Therefore, we considered this virus as a new species. The MraTV2 sequences shared more than 80% identity with DTtV1 (UYL95681.1), which was identified in a tick metagenome. We have therefore hypothesised that the tick could harbor *Malassezia* on its surface or in its digestive system, and that this virus comes from *Malassezia* rather than from the tick itself. However, this hypothesis cannot be tested because the metagenomic data are not publicly available. Our RNA-Seq approach also successfully identified both RdRp and CP sequences of MrV40L in the *M. restricta* meta transcriptomic data of the strain from which this virus had been characterised [4]. Our study has two main limitations. On the one hand, the putative mycovirus genomes detected in this study might have been derived from mycoviruses that have been integrated in the *Malassezia* genome. On the other hand, the 86% *Malassezia* transcriptomes in which we did not detect any virus-associated contigs might be infected by unknown mycoviruses. Indeed, omics approaches cannot detect unknown viruses that are too dissimilar from currently known viral sequences.

The phylogenetic analysis of the detected RdRP and CP protein sequences showed that the amino acid sequences of the three newly identified *Totivirus*-like contigs clustered with unclassified *Totiviridae*, unclassified *Totivirus*, and the genus *Totivirus.* Regarding the RdRP amino acid sequences of the two predicted *Totivirus*-like contigs, MgaTV1 and MraTV1 clustered with an unclassified *Totivirus* that is known to infect sea cucumbers (*Holothuroidea* and *Echinodermata*) [31] and constituted two distinct subclades closely related with totiviruses infecting *Erysiphe necator*, the agent of powdery mildew in grapes.

Regarding ORFs encoding the CP, those of MgaTV1 clustered with the unclassified *Totivirus,* totivirus sp., and those of MraTV1 clustered with an unclassified *Totiviridae* that has been detected in a tick metagenome. CP sequences of MraTV2 clustered with Dali Totiv tick virus 1 which was also found in a tick metagenome. Both RdRP and CP amino acid sequences of MrV40L from *M. restricta* were closely related and shared significant similarity with MrV40L (QJA42331.1 and QJA42330.1, respectively) identified by Park et al. [4]. We also identified, in the RdRP sequences of MgaTV1 and MraTV1, eight conserved motifs similar to the one to eight motifs identified by Bruenn [28] (Figure 3). We retrieved only two motifs in RdRP of MrV40L in contrast to Park et al. [4], because the size of the MrV40L RdRP (160 aa) was smaller than that of the previously reported Mrv40 RdRP (862 aa). We described eight novel conserved motifs in the CP sequences of MgaTV1, MraTV1, and MraTV2, and most of their closely related species (Figure S1).

The presence of *Malassezia* associated-mycoviruses identified in this study raises questions about their potential influence on *Malassezia* virulence. Viruses from the genus *Totivirus* are currently known to infect a variety of fungi, in some of which a decrease in virulence has been observed [32]. In addition, recent studies have reported the effects of *Totiviridae* mycoviruses on *Malassezia* yeast. They showed that MsMV1 induces the overexpression of transcription factors and ribosomal genes and a higher level of IFN-β expression in cultured macrophages. Meanwhile, MrV40L has also been shown to upregulate TLRs and cytokines and to have an impact on several physiological processes in *M. restricta* [4,17]. It is possible that one or more mycoviruses identified in this study might decrease or increase the virulence of some *Malassezia* species. The identification of a virus by only an in silico approach does not demonstrate that the virus infects a living organism. This must be confirmed by other methods.

In conclusion, this study confirms the presence of mycoviruses in *M. globosa* and *M. restricta*, which are among of the most abundant yeast of the human skin mycobiome. Additionally, we report for the first time the detection of three novel *Totiviridae* signatures in *Malassezia* transcriptomes. These findings expand our knowledge about the diversity of mycoviruses in this yeast of clinical importance.

## Data Availability

All data were submitted at EMBL under the bioproject number PRJEB62036.

## Supplementary Materials

The following supporting information can be downloaded at: https://www.mdpi.com/article/10.3390/v15061368/s1, Figure S1: Conserved motifs identified in the CP domain of the genus *Totivirus*; Table S1: Summary of *Malassezia* transcriptomes used in this study; Table S2: Candidate mycovirus-like sequences from *Malassezia* RNA-seq datasets; Table S3: Comparison of Chargaff’s Ratio (GC%) between identified sequences and closely related species; Table S4: Functional annotation of candidate viral ORFs using PHYRE2, Table S5: List of accession numbers of closely-related *Totiviruses* used for the phylogenetic tree reconstruction.
