# Supplementary material for: Tracking Mycoviruses in Public RNAseq Datasets of Malassezia: Three Original Totiviruses Revealed
Source: Viruses. 2023 Jun 13;15(6):1368. doi: 10.3390/v15061368 (PMC10305220; doi:10.3390/v15061368)
Supplement: Supplementary file 1 [file viruses-15-01368-s001.zip › Supplementary Figure S1.pptx]

## Slide 1
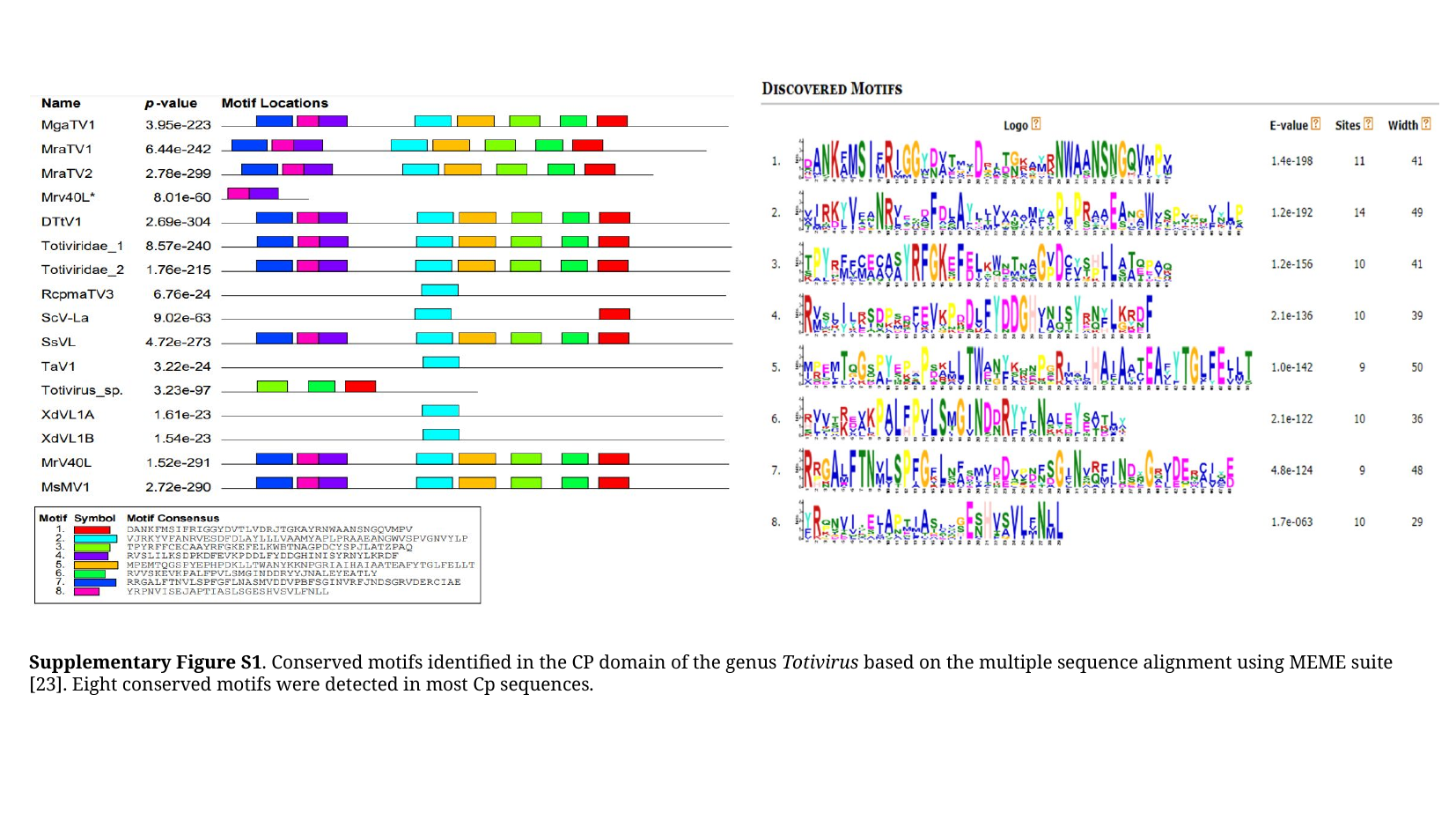

Supplementary Figure S1. Conserved motifs identified in the CP domain of the genus Totivirus based on the multiple sequence alignment using MEME suite [23]. Eight conserved motifs were detected in most Cp sequences.
