# Supplementary material for: Tracking Mycoviruses in Public RNAseq Datasets of Malassezia: Three Original Totiviruses Revealed
Source: Viruses. 2023 Jun 13;15(6):1368. doi: 10.3390/v15061368 (PMC10305220; doi:10.3390/v15061368)
Supplement: Supplementary file 1 [file viruses-15-01368-s001.zip › Supplementary Table S2.pdf]

Supplementary Table S2. Candidate mycovirus-like sequences from *Malassezia* RNA-seq datasets.

BLASTX / BLASTP													
Fungal Host	BioProject and SRA ID(s)	Citation for Data	Contig (ORF)	Length(aa)	Hit with highest score in Blastp vs NR(NCBI)	Segment Number	Length(aa)	Accession	Protein ID	Query Cover	% aa identity	Virus Family	VirusClass
Malassezia globosa	**PRJNA286710** (SRR2073303)	(GIS)	DN4160_c0_g3_i1.p1	767	Erysiphe necator associated totivirus 7	1	796	QJW70337	RdRp	99%	46.87%	*Totiviridae*	dsRNA
			DN4160_c0_g2_i1.p1	305	Totiviridae sp.	1	693	WAK77569.1	capsid protein	96%	41.22%		
			DN4160_c0_g3_i1.p2	260		2				95%	37.85%		
	(SRR2072161)	(GIS)	DN603_c1_g3_i1.p1	753	Erysiphe necator associated totivirus 7	1	796	QJW70337	RdRp	99%	47.59%	*Totiviridae*	dsRNA
			DN603_c1_g2_i1.p1	419	Totiviridae sp.	1	693	WAK77569.1	capsid protein	97%	41.84%		
			DN603_c1_g1_i1.p1	428		2				99%	42.19%		
			DN603_c1_g3_i1.p2	269		3				98%	36.43%		
	(SRR2072162)	(GIS)	DN1126_c0_g1_i1.p1	767	Erysiphe necator associated totivirus 7	1	796	QJW70337	RdRp	99%	46.87%	*Totiviridae*	dsRNA
			DN1126_c0_g1_i1.p2	267	Totiviridae sp.	1	693	WAK77569.1	capsid protein	92%	37.85%		
			DN1126_c0_g3_i1.p1	433		2				95%	42.17%		
			DN1126_c0_g3_i2.p1	443		3				96%	42.19%		
	(SRR2073318)	(GIS)	DN864_c0_g3_i1.p1	716	Erysiphe necator associated totivirus 7	1	796	QJW70337	RdRP	99%	47.89%	*Totiviridae*	dsRNA
			DN864_c0_g2_i1.p1	419	Totiviridae sp.	1	693	WAK77569.1	capsid protein	97%	41.89%		
			DN864_c0_g4_i1.p1	434		2				98%	42.19%		
	(SRR2072153)	(GIS)	DN461_c1_g1_i1.p1	767	Erysiphe necator associated totivirus 7	1	796	QJW70337	RdRP	99%	46.87%	*Totiviridae*	dsRNA
			DN461_c1_g1_i1.p2	688	Totiviridae sp.	1	693	WAK77569.1	capsid protein	98%	40.00%		
	(SRR2072157)	(GIS)	DN3320_c1_g2_i1.p1	767	Erysiphe necator associated totivirus 7	1	796	QJW70337	RdRp	99%	46.87%	*Totiviridae*	dsRNA
			DN3320_c1_g1_i1.p1	262	Totiviridae sp.	1	693	WAK77569.1	capsid protein	93%	40.98%		
			DN3320_c1_g2_i1.p2	688		2				98%	40.00%		
	(SRR2072163)	(GIS)	DN765_c1_g1_i1.p1	723	Erysiphe necator associated totivirus 7	1	796	QJW70337	RdRp	100%	47.51%	*Totiviridae*	dsRNA
			DN765_c1_g2_i1.p2	670		2				95%	50.55%		
			DN765_c1_g1_i1.p2, DN765_c1_g2_i1.p1	688	Totiviridae sp.	2	693	WAK77569.1	capsid protein	98%	40.00%		
	(SRR2072150)	(GIS)	DN527_c1_g1_i1.p1	767	Erysiphe necator associated totivirus 7	1	796	QJW70337	RdRp	99%	46.87%	*Totiviridae*	dsRNA
			DN527_c1_g1_i1.p2	688	Totiviridae sp.	1	693	WAK77569.1	capsid protein	98%	40.00%		
	(SRR2072151)	(GIS)	DN616_c2_g1_i1.p1, DN616_c2_g2_i1.p1	767	Erysiphe necator associated totivirus 7	2	796	QJW70337	RdRp	99%	46.87%	*Totiviridae*	dsRNA

			DN616_c2_g2_i1.p2	688	Totiviridae sp.	1	693	WAK77569.1	capsid protein	98%	40.00%		
	(SRR2072152)	(GIS)	DN94_c1_g1_i1.p1	767	Erysiphe necator associated totivirus 7	1	796	QJW70337	RdRp	99%	46.87%	*Totiviridae*	dsRNA
			DN94_c1_g1_i1.p2	688	Totiviridae sp.	1	693	WAK77569.1	capsid protein	98%	40.00%		
	(SRR2072154)	(GIS)	DN271_c0_g1_i1.p1	767	Erysiphe necator associated totivirus 7	1	796	QJW70337	RdRp	99%	46.87%	*Totiviridae*	dsRNA
			DN271_c0_g1_i1.p2	688	Totiviridae sp.	1	693	WAK77569.1	capsid protein	98%	40.00%		
	(SRR2072158)	(GIS)	DN1612_c1_g2_i1.p1	742	Erysiphe necator associated totivirus 7	1	796	QJW70337	RdRp	99%	47.57%	*Totiviridae*	dsRNA
			DN1612_c1_g3_i1.p1	420	Totiviridae sp.	1	693	WAK77569.1	capsid protein	98%	42.17%		
			DN1612_c1_g1_i1.p1	438		2				97%	42.19%		
	(SRR2072159)	(GIS)	DN718_c0_g2_i1.p1	767	Erysiphe necator associated totivirus 7	1	796	QJW70337	RdRp	97%	46.87%	*Totiviridae*	dsRNA
			DN718_c0_g2_i1.p2	688	Totiviridae sp.	1	693	WAK77569.1	capsid protein	98%	40.00%		
			DN718_c0_g1_i1.p1	159		2				97%	42.58%		
	(SRR2072129)	(GIS)	DN172_c0_g1_i1.p1	767	Erysiphe necator associated totivirus 7	1	796	QJW70337	RdRp	99%	46.87%	*Totiviridae*	dsRNA
			DN172_c0_g1_i1.p2	688	Totiviridae sp.	1	693	WAK77569.1	capsid protein	98%	40.00%		
			DN172_c0_g2_i1.p1	610		*2*				*99%*	*41.12%*		
	(SRR2072130)	(GIS)	DN4202_c0_g2_i1.p1, DN4202_c0_g1_i1.p1	767	Erysiphe necator associated totivirus 7	2	796	QJW70337	RdRp	99%	46.87%	*Totiviridae*	dsRNA
			DN4202_c0_g2_i1.p2, DN4202_c0_g1_i1.p2	688	Totiviridae sp.	2	693	WAK77569.1	capsid protein	98%	40.00%		
	(SRR2072131)	(GIS)	DN3768_c1_g1_i1.p1	767	Erysiphe necator associated totivirus 7	1	796	QJW70337	RdRp	99%	46.87%	*Totiviridae*	dsRNA
			DN3768_c1_g1_i1.p2	688	Totiviridae sp.	1	689	WAK77569.1	capsid protein	98%	40.00%		
	(SRR2072147)	(GIS)	DN3904_c0_g3_i1.p1	767	Erysiphe necator associated totivirus 7	1	796	QJW70337	RdRp	99%	46.87%	*Totiviridae*	dsRNA
			DN3904_c0_g2_i1.p1	438	Totiviridae sp.	1	693	WAK77569.1	capsid protein	97%	42.19%		
			DN3904_c0_g1_i1.p1	417		*2*				98%	42.17%		
	(SRR2072148)	(GIS)	DN5042_c0_g3_i1.p1	767	Erysiphe necator associated totivirus 7	1	796	QJW70337	RdRp	99%	46.87%	*Totiviridae*	dsRNA
			DN5042_c0_g3_i1.p2	267	Totiviridae sp.	1	693	WAK77569.1	capsid protein	92%	37.85%		
			DN5042_c0_g2_i1.p1	430		2				99%	42.19%		
			DN5042_c0_g1_i1.p1	417		3				98%	42.17%		
	(SRR2072149)	(GIS)	DN3496_c1_g1_i1.p1	767	Erysiphe necator associated totivirus 7	1	796	QJW70337	RdRp	99%	46.87%	*Totiviridae*	dsRNA
			DN3496_c1_g1_i1.p2	688	Totiviridae sp.	1	693	WAK77569.1	capsid protein	98%	40.00%		
	(SRR2072160)	(GIS)	DN1036_c0_g1_i1.p1	767	Erysiphe necator associated totivirus 7	1	796	QJW70337	RdRp	99%	46.87%	*Totiviridae*	dsRNA
			DN1036_c0_g2_i1.p1	262	Totiviridae	1	693	WAK77569.1	capsid	93%	40.98%		

			DN1036_c0_g1_i1.p2	688	sp.	2			protein	98%	40.00%		
	(SRR2072165)	(GIS)	DN1307_c0_g1_i1.p1	767	Erysiphe necator associated totivirus 7	1	796	QJW70337	RdRp	99%	46.87%	*Totiviridae*	dsRNA
			DN1307_c0_g1_i1.p2	688	Totiviridae sp.	1	693	WAK77569.1	capsid protein	98%	40.00%		
			DN1307_c0_g2_i1.p1	420		2				98%	42.17%		
	(SRR2073308)	(GIS)	DN1099_c1_g2_i1.p1	764	Erysiphe necator associated totivirus 7	1	796	QJW70337	RdRp	99%	46.92%	*Totiviridae*	dsRNA
			DN1099_c1_g3_i1.p1	262	Totiviridae sp.	1	693	WAK77569.1	capsid protein	93%	40.98%		
			DN1099_c1_g1_i2.p1	437		2				97%	42.19%		
			DN1099_c1_g1_i1.p1	420		3				98%	42.17%		
	(SRR2073314)	(GIS)	DN5782_c1_g1_i1.p1	767	Erysiphe necator associated totivirus 7	1	796	QJW70337	RdRp	99%	46.87%	*Totiviridae*	dsRNA
			DN5782_c1_g1_i1.p2	688	Totiviridae sp.	1	693	WAK77569.1	capsid protein	98%	40.00%		
			DN5782_c1_g2_i1.p1	420		2				98%	42.17%		
	(SRR2073323)	(GIS)	DN1391_c0_g1_i1.p1	767	Erysiphe necator associated totivirus 7	1	796	QJW70337	RdRp	99%	46.87%	*Totiviridae*	dsRNA
			DN1391_c0_g2_i1.p1	159	Totiviridae sp.	1	693	WAK77569.1	capsid protein	97%	42.58%		
			DN1391_c0_g1_i1.p2	688		2				98%	40.00%		
	(SRR2073327)		DN910_c0_g3_i1.p1	767	Erysiphe necator associated totivirus 7	1	796	QJW70337	RdRp	99%	46.87%	*Totiviridae*	dsRNA
			DN910_c0_g2_i1.p1	429	Totiviridae sp.	1	693	WAK77569.1	capsid protein	97%	41.89%		
			DN910_c0_g1_i1.p1	446		1				97%	42.19%		
Malassezia restricta	**PRJNA577935** (SRR10294524)	(SNU)	DN1447_c0_g1_i2.p1	160	Malassezia restricta virus MrV40L	1	862	QJA42331	RdRp	99%	100%	*Totiviridae*	dsRNA
			DN1206_c1_g9_i2.p1	120	Malassezia restricta virus MrV40L	1	689	QJA42330	capsid protein	100%	100%		
	**PRJNA593855** (SRR10597458)	(SNU)	DN296_c0_g1_i1.p1 , DN296_c0_g2_i1.p1	691	Totiviridae sp.	2	790	WAK77294.1	polyprotein	99%	49.64%	*Totiviridae*	dsRNA
			DN296_c0_g2_i1.p2	658	Totiviridae sp.	1	691	WAK77450.1	capsid protein	98%	43.16%		
			DN296_c0_g1_i1.p2	598		2				99%	42.24%		
			DN1846_c0_g1_i1.p1	466	Dali Totiv tick virus 1	1	690	UYL95681.1	capsid protein	100%	84.28%		
			DN1846_c0_g2_i1.p1	378		2				100%	84.62%		
	(SRR10597459)	(SNU)	DN1827_c0_g1_i1.p1	433	Totiviridae sp.	1	790	WAK77294.1	polyprotein	93%	60.81%	*Totiviridae*	dsRNA
			DN1827_c0_g2_i1.p1	501		2				99%	57.06%		
			DN4_c0_g2_i1.p1	510	Totiviridae sp.	1	691	WAK77450.1	capsid protein	96%	42.80%		
			DN122_c0_g2_i1.p1	587	Dali Totiv tick virus 1	1	690	UYL95681.1	capsid protein	100%	83.79%		
			DN122_c0_g2_i2.p1	562		2				98%	84.2%		
