# Supplementary material for: Tracking Mycoviruses in Public RNAseq Datasets of Malassezia: Three Original Totiviruses Revealed
Source: Viruses. 2023 Jun 13;15(6):1368. doi: 10.3390/v15061368 (PMC10305220; doi:10.3390/v15061368)
Supplement: Supplementary file 1 [file viruses-15-01368-s001.zip › Supplementary Table S3_S4_S5.pdf]

Supplementary Table S3. Comparison of Chargaff's Ratio (GC%) between identified sequences and closely related species.

Virus-associated contigs	Chargaff's Ratio GC (%) of virus associated contigs	Chargaff's Ratio GC (%) of the closest viral species
MgaTV1_RdRP	49.2	EnaT7<br>41.9
MgaTV1_CP	49.5	Totiviridae sp.<br>49.8
MraTV1_CP	48.3	Totiviridae sp.<br>46.2
MraTV1_polyprotein	50.2	
MraTV2_CP	44	Dali Totiv tick virus 1<br>42.5
MrV40L_RdRP	45.4	MrV40L<br>46.3
MrV40L_CP	43.7	

Supplementary Table S4. Functional annotation of candidate viral ORFs using PHYRE2.

Virus associated ORFs	Best hits	Confidence	%id
MgaTV1_RdRP	**PDB header**:viral protein, replication<br>**Chain**: A: **PDB Molecule**:potential rna-dependent rna polymerase	100.0	20
MgaTV1_CP	**Fold**: L-A virus major coat protein<br>**Superfamily**: L-A virus major coat protein	100.0	22
MraTV1_CP	**Fold**: DNA/RNA polymerases<br>**Family**: RNA-dependent RNA-polymerase	100.0	23
MraTV2_CP	**Fold**: L-A virus major coat protein<br>**Superfamily**: L-A virus major coat protein	82.2	22
MraTV1_Polyprotein	**PDB header**:hydrolase<br>**Chain**: A: **PDB Molecule**:rna-directed rna polymerase	99.7	17
MrV40L_RdRP	**Fold**: DNA/RNA polymerases<br>**Family**: RNA-dependent RNA-polymerase	95.6	27
MrV40L_CP	**Fold**: L-A virus major coat protein<br>**Superfamily**: L-A virus major coat protein	90.3	34

Supplementary Table S5. List of accession numbers of closely-related Totiviruses used for the phylogenetic tree reconstruction (RDRP and CP).

Genus	Species (strain abbreviation)	RNA-dependent RNA-polymerase (RdRP)	Capsid protein (CP)
Totivirus	Dali Totiv tick virus 1 (DTtV1)	-	UYL95681.1
	Delisea pulchra totivirus IndA (DpT_IndA)	AMB17468	-
	Erysiphales associated mitovirus 1 (EaM1)	QIP68035.1	-
	Erysiphales associated totivirus 1 (EnaT1)	QIP68044	-
	Erysiphe necator associated totivirus 6 (EnaT6)	QJW70335	-
	Erysiphe necator associated totivirus 7 (EnaT7)	QJW70337.1	-
	Erysiphe necator associated totivirus 8 (EnaT8)	QJW70339	-
	Erysiphales associated totivirus 13 (EnaT13)	QIP68067	-
	Phakopsora pachyrhizi mycovirus (PpM)	ALO81041.1	QED43015.1
	Red clover powdery mildew-associated totivirus 3 (RcpmaTV3)	YP_009182181.1	YP_009182180
	Saccharomyces cerevisiae virus L-BC (La) (ScV-La)	NP_042581	NP_042580
	Scheffersomyces segobiensis virus L (SsVL)	YP_009507831	YP_009507830
	Totiviridae sp.1	WAK77294.1	-
	Totiviridae sp.2	-	WAK77569.1
	Totiviridae sp.3	-	WAK77450.1
	Totivirus sp.	QOQ37359	QOQ37361.1
	Tuber aestivum virus 1 (TaV1)	YP_009507833.1	YP_009507832
	Xanthophyllomyces dendrorhous virus L1A (XdVL1A)	YP_007697651.1	AFH09411.1
	Xanthophyllomyces dendrorhous virus L1B (XdVL1B)	YP_009507835.1	YP_009507834.1
Giardiavirus	Drosophila melanogaster totivirus (DTV)	YP_003289293.1	UFT26918.1
	Golden shiner totivirus (GSTV)	YP_009256209.1	-
	Omono River virus (OMRV)	AUI11418.1	AUI11420.1
	Tianjin totivirus (ToV)	YP_005454251.1	YP_005454250.1
Victorivirus	Gremmeniella abietina virus L1 (GaRV-L1)	AAK11656.1	-
	Helicobasidium mompa totivirus 1-17 (HmV-17)	BAC81754.1	-

	Macrophomina phaseolina victorivirus 1 (MpV1)	QKI37143.1	-
	Magnaporthe oryzae virus 1 (MoV1)	BAD60833.1	-
Unclassified *Totivirus*	Malassezia restricta virus MrV40L (MrV40L)	QJA42331.1	QJA42330.1
	Malassezia sympodialis mycovirus (MsMV1)	QNJ34609	QNJ34608.1
Reoviridae	Human rotavirus A (ROTHA)	ACR22792	AIQ80953.1
